# Primary Disruption of the Memory-Related Subsystems of the Default Mode Network in Alzheimer’s Disease: Resting-State Functional Connectivity MRI Study

**DOI:** 10.3389/fnagi.2018.00344

**Published:** 2018-10-31

**Authors:** Huihui Qi, Hao Liu, Haimeng Hu, Huijin He, Xiaohu Zhao

**Affiliations:** ^1^Department of Medical Imaging, Tongji Hospital, Tongji University School of Medicine, Tongji University, Shanghai, China; ^2^Department of Imaging, Huashan Hospital, Fudan University, Shanghai, China; ^3^Department of Imaging, The Fifth People’s Hospital of Shanghai, Fudan University, Shanghai, China; ^4^Department of Imaging, Shanghai Tongji Hospital, Shanghai, China

**Keywords:** resting-state fMRI, default network, subsystems, functional connectivity, Alzheimer’s disease

## Abstract

**Background:** Recent studies have indicated that the default mode network (DMN) comprises at least three subsystems: The medial temporal lobe (MTL) and dorsal medial prefrontal cortex (DMPFC) subsystems and a core comprising the anterior MPFC (aMPFC) and posterior cingulate cortex (PCC). Additionally, the disruption of the DMN is related to Alzheimer’s disease (AD). However, little is known regarding the changes in these subsystems in AD, a progressive disease characterized by memory impairment. Here, we performed a resting-state functional connectivity (FC) analysis to test our hypothesis that the memory-related MTL subsystem was predominantly disrupted in AD.

**Method:** To reveal specific subsystem changes, we calculated the strength and number of FCS in the DMN intra- and inter-subsystems across individuals and compared the FC of the two groups. To further examine which pairs of brain regional functional connections contributed to the subsystem alterations, correlation coefficients between any two brain regions in the DMN were compared across groups. Additionally, to identify which regions made the strongest contributions to the subsystem changes, we calculated the regional FC strength (FCS), which was compared across groups.

**Results:** For the intra-subsystem, decreased FC number and strength occurred in the MTL subsystem of AD patients but not in the DMPFC subsystem or core. For the inter-subsystems, the AD group showed decreased FCS and number between the MTL subsystem and PCC and a decreased number between the PCC and DMPFC subsystem. Decreased inter-regional FCS were found within the MTL subsystem in AD patients relative to controls: The posterior inferior parietal lobule (pIPL) showed decreased FC with the hippocampal formation (HF), parahippocampal cortex (PHC) and ventral MPFC (vMPFC). Decreased inter-regional FCS of the inter-subsystems were also found in AD patients: The HF and/or PHC showed decreased FC with dMPFC and TPJ, located in the DMPFC subsystem, and with PCC. AD patients also showed decreased FC between the PCC and TLC of the dMPFC subsystem. Furthermore, the HF and PHC in the MTL subsystem showed decreased regional FCS.

**Conclusion:** Decreased intrinsic FC was mainly associated with the MTL subsystem of the AD group, suggesting that the MTL subsystem is predominantly disrupted.

## Introduction

Since its name was proposed more than a decade ago (Raichle et al., 2001), the default mode network (DMN) has been characterized by its high level of metabolic activity during passive states and low activity in externally directed conditions (Shulman et al., 1997; Gusnard and Raichle, 2001; Raichle et al., 2001). Additionally, the robust activity correlations within the DMN at rest and during task performance have been confirmed in many papers (Greicius et al., 2003; Fox et al., 2005; Buckner et al., 2008; Grigg and Grady, 2010; Spreng and Grady, 2010; Allen et al., 2011). Although the precise function of the DMN is still debated, most literature has revealed that the DMN shows increased activity not only during rest but also during tasks, as long as experimental conditions involve aspects of self-generated thought (Andrews-Hanna et al., 2014), such as autobiographical memory (Svoboda et al., 2006; Spreng and Grady, 2010), future projection (Addis et al., 2007), mind wandering (Christoff et al., 2009; Smallwood et al., 2013), and social cognition (Mar, 2010). These features of self-generated thought suggest that they comprise multiple-component processes (Andrews-Hanna et al., 2014), which serve to prepare for upcoming events (Baird et al., 2011), form a sense of self-identity and continuity across time (Smallwood et al., 2011; Prebble et al., 2013), and navigate the social world (Immordino-Yang et al., 2012; Mar et al., 2012; Ruby et al., 2013). Recently, converging evidence has revealed that the DMN has a parallel level of complexity in functional-anatomical organization to support multiple-component processes of self-generated thought (Buckner et al., 2008; Sestieri et al., 2011; Andrews-Hanna, 2012; Kim, 2012; Roy et al., 2014). Andrews-Hanna et al. (2010b) explored the intrinsic functional organization of the DMN by using functional connectivity magnetic resonance imaging (fcMRI), graph analysis and hierarchical clustering techniques. The authors suggested that the DMN comprised two distinct subsystems: a dorsal medial prefrontal cortex (DMPFC) subsystem consisting of the dMPFC, the temporal parietal junction (TPJ), the lateral temporal cortex (LTC), and the temporal pole (TempP), and an MTL subsystem comprising the ventral medial prefrontal cortex (vMPFC), the posterior inferior parietal lobule (pIPL), the retrosplenial cortex (Rsp), the hippocampal formation (HF), and the parahippocampal cortex (PHC). Both subsystems converge on a core that includes the anterior MPFC (aMPFC) and the posterior cingulate cortex (PCC). The authors then proved that the two subsystems had relatively distinct functions by designing an experiment. The DMPFC subsystem was preferentially activated during decisions about one’s present situation or mental state, and the MTL subsystem was selectively activated during self-relevant predictions about one’s future.

Previous work has consistently shown that compared with healthy subjects, patients with Alzheimer’s disease (AD) have reduced FC within the DMN when at rest (Greicius et al., 2004; Rombouts et al., 2005; Celone et al., 2006; Wang K. et al., 2006) and less pronounced deactivation during cognitive tasks (Lustig et al., 2003; Celone et al., 2006). In addition, several studies of cortical hubs that performed functional and structural connectivity analysis combined with graph theoretical analysis revealed that most of the hubs are located within the DMN, such as the PCC, the medial prefrontal cortex (MPFC), and the HF (Hagmann et al., 2008; Buckner et al., 2009; Yu et al., 2017). It has been suggested that these cortical hubs in the DMN are preferentially affected in AD (Yu et al., 2017). The abovementioned studies have suggested that DMN impairment is related to AD. Moreover, although several studies have found that reductions in FC only exist in some regions, such as the PCC and the medial temporal lobe (MTL), in patients with AD (Zhou et al., 2008; Bai et al., 2009), other studies have reported widespread reductions in FC as the disease progresses (Damoiseaux et al., 2012). AD is a progressive dementia, and the predominant symptom is memory impairment, although disturbances in other cognitive functions also occur (Krajcovicova et al., 2014). Based on the above findings, distinct subsystems in the DMN engage in differential cognitive processes involving memory construction and self-oriented cognition, and these cognitive processes are influenced by AD (Greicius et al., 2004; Buckner et al., 2008, 2009; Zhou et al., 2010; Menon, 2011). Therefore, we hypothesized that the memory-related MTL subsystem was predominantly disrupted in AD and that other subsystems in the DMN may be disrupted to a certain degree. To test these hypotheses, rs-fMRI was performed on 24 patients with mild to moderate AD and on 27 healthy elderly subjects. Eleven regions of interest (ROIs) in the DMN subsystem for FC analysis were selected based on published data by Andrews-Hanna et al. (2010b). The DMN FC matrices were constructed by estimating the Pearson correlation between the time series of pairs of ROIs in the subsystems. To reveal the specific disrupted subsystem, we analyzed the strength and number of FCS in the intra- and inter-subsystems of the DMN. Then, to examine which pairs of regional FCS contributed to changes in the subsystems, each pair of correlation coefficients in the DMN was compared across groups.

In the present study, a regional FCS analysis was also used to identify regions that made the strongest contributions to the changes in the subsystems. Regional FCS is defined as the mean of the correlation strength of one ROI with all other target regions in a brain network and is referred to as the “degree centrality” of a weighted network in graph theory (Buckner et al., 2009; Zuo et al., 2012; Wang L. et al., 2013). Brain ROIs with higher FCS values usually indicate their central roles in the functional integrity of the brain network (Wang L. et al., 2013).

**Table 1:** Demographics and clinical information.

Characteristics	HC (*n* = 27)	AD (*n* = 24)	*P*
Age	63.74 ± 7.80	67.54 ± 10.48	0.146^a^
Female/Male	11/16	13/11	0.406^b^
MMSE	28.84 ± 1.19	21.46 ± 1.67	<0.001^a^

*Data are presented as the mean ± standard deviation (SD). ^a^The P-value was obtained using a two-sample t-test. ^b^The P-value was obtained using the Pearson chi-square test.*

## Materials and Methods

### Participants

A total of 55 subjects were enrolled from Shanghai Huashan Hospital. All participants were categorized into a control group (*n* = 27) and an AD patient group (*n* = 28). AD patients were diagnosed by a qualified neurologist using criteria for amnestic AD (Fennema-Notestine et al., 2009), with mini-mental state examination (MMSE) scores between 12 and 27 (inclusive) and clinical dementia rating (CDR) scores of 1 or 2. The control groups had MMSE scores between 26 and 30 (inclusive) and CDR scores of 0. The data of 4 subjects (4 patients with AD) were excluded due to excessive motion, severe brain atrophy, hydrocephalus or large areas of cerebral infarction. Details regarding the clinical and demographic data of the remaining 51 subjects are shown in Table 1. There were no significant differences in terms of sex or age between the two groups.

### Image Acquisition and Preprocessing

All subjects underwent whole-brain MRI scanning on a 3.0T SIEMENS Verio. Resting-state BOLD functional MRI data were collected using an echo-planar imaging (EPI) sequence. The scanning parameters were TR = 2000 ms, TE = 35 ms, FOV = 25.6 cm × 25.6 cm, flip angle = 90°, matrix = 256 × 256, slices = 33, thickness = 4 mm, and gap = 4 mm.

Unless specifically stated otherwise, all of the preprocessing was performed using statistical parametric mapping (SPM8^1^). The first 5 images were discarded in consideration of magnetization equilibrium. The remaining 155 images were corrected for the acquisition time delay among different slices, and then, the images were realigned to the first volume for head-motion correction. The fMRI images were further spatially normalized to the Montreal Neurological Institute (MNI) EPI template and resampled to a 2-mm cubic voxel. Several sources of spurious variance, including the estimated motion parameters, the linear drift, and the average time series in the cerebrospinal fluid and white matter regions, were removed from the data through linear regression. Finally, temporal bandpass filtering (0.03–0.06 HZ) was performed to reduce the effects of low-frequency drift and high-frequency noise (Liu et al., 2008; Zhang et al., 2012). The time course of head motion was obtained by estimating the translations in each direction and the rotations in angular motion about each axis for each of the 155 consecutive volumes. All of the subjects included in this study exhibited a maximum displacement of less than 3 mm (smaller than the size of a voxel in plane) at each axis and an angular motion of less than 3 for each axis. Data from two subjects were excluded due to excessive motion.

**Table 2:** Regions of interest (ROIs) within the DMN subsystems for functional connectivity analysis from Andrews-Hanna et al. (2010b).

Region	Abbreviation	*x*	*y*	*z*
Core region				
Anterior medial prefrontal cortex	aMPFC	−6	52	−2
Posterior cingulate cortex	PCC	−8	−56	26
DMPFC subsystem				
Dorsal medial prefrontal cortex	dMPFC	0	52	26
Temporal parietal junction	TPJ	−54	−54	28
Lateral temporal cortex	LTC	−60	−24	−18
Temporal pole	TempP	−50	14	−40
MTL subsystem				
Ventral medial prefrontal cortex	vMPFC	0	28	−18
Posterior inferior parietal lobule	pIPL	−44	−74	32
Retrosplenial cortex	Rsp	−14	−52	8
Parahippocampal cortex	PHC	−28	−40	−12
Hippocampal formation	HF	−22	−20	−26

*Coordinates are based on the Montreal Neurological Institute coordinate system.*

### Functional Connectivity Analysis

fcMRI was performed using REST^2^ (Song et al., 2011) to extract the time series for each ROI (spherical radius of 4 mm) (Table 2) by averaging the time courses of all voxels within an ROI. For each subject, we first calculated a Pearson’s correlation and the significance level (i.e., *p*-value) between all given ROIs. Then, we obtained an 11 × 11 symmetric correlation matrix and the corresponding *p*-value matrix for each subject. To eliminate unreliable correlations, we retained only those correlations whose corresponding *p*-values met a statistical threshold *p* < 0.001 (0.05/55, Bonferroni correction); otherwise, we set the correlations to zero (Cruse et al., 2011; Wang et al., 2016). Then, correlation coefficients in a weighted 11 × 11 FC matrix were converted into *z*-values with the application of Fisher’s r-to-z transformation. Fisher’s Z-transformed correlation matrix was used for the subsequent analysis of each subject. The strength of the intra-subsystem FCS was defined as the total and mean of all positive-only inter-regional FCS within the selected subsystem, while the strength of the inter-subsystem FCS was defined as the total and mean of the positive-only FCS between any two ROIs of the two selected subsystems. In this paper, we calculated the strength of the FCS of the intra- and inter-subsystems of each subject and compared them across groups using two-sample *t*-tests. We also analyzed the differences in the numbers of positive-only FCS of the intra- and inter-subsystems between two groups using two-sample *t*-tests. Notably, to explore the specific changes in FC between the core regions and subsystems, we calculated the number and strength of FCS between the two core regions and subsystems separately. To examine which pairs of brain regional FCS contributed to the alterations in the subsystems of the DMN in AD, the correlation coefficients between any two ROIs in the DMN were also compared across groups using two-sample *t*-tests with false discovery rate (FDR) correction. We also listed the results with *p*-values less than 0.05, 0.01, and 0.001 to find possible differences in physiological significance. Furthermore, to identify which regions made the highest contributions to the changes in the subsystems, we compared the regional FCS across groups by using two-sample *t*-tests. The FCS of a given region was calculated using the following equation (Buckner et al., 2009; Zuo et al., 2012; Wang L. et al., 2013):

$$\mathrm{FCS}(i)=\frac{1}{N-1}\sum_{j=1,j\neq i}^{N}z_{\mathrm{ij}},r_{\mathrm{ij}}>r_{0}$$

where *N* is the number of ROIs in the DMN (here *N* = 11), *r*_ij_ *z*_ij_ is the Fisher Z-transformed correlation coefficient, *r*_ij_is the correlation coefficient between ROI i and ROI j, and r0 is a correlation threshold that was used to eliminate weak correlations possibly derived from noise (here, *r*0 = 0). The difference in regional FCS between the two groups revealed regions that were disrupted in patients with AD.

### The Effects of Different Analysis Strategies

In our study, an FC matrix included positive and negative connections. In the positive-only strategy, we analyzed FC matrices by using the positive-only inter-regional FCS. For example, we assumed that no inter-regional FC existed if the inter-regional FC was non-positive. Given the controversies in the treatment of negative correlations in rs-fMRI network studies (Fox et al., 2009; Wang et al., 2011; Dai et al., 2015), we also calculated the strengths and numbers of positive and negative FCS of the intra- and inter-subsystems for the two groups and performed an FCS analysis including both positive and negative (absolute values) connections to assess the stability of our findings.

## Results

### Group Differences in the Strengths and Numbers of FCS of the Subsystems and Core

To explore specific changes in the subsystems in AD patients, the total and mean FC strengths of the intra- and inter-subsystems of the DMN were calculated. Different analysis strategies consistently produced the same results. In this study, we show the results of the positive-only strategy.

**FIGURE 1:**
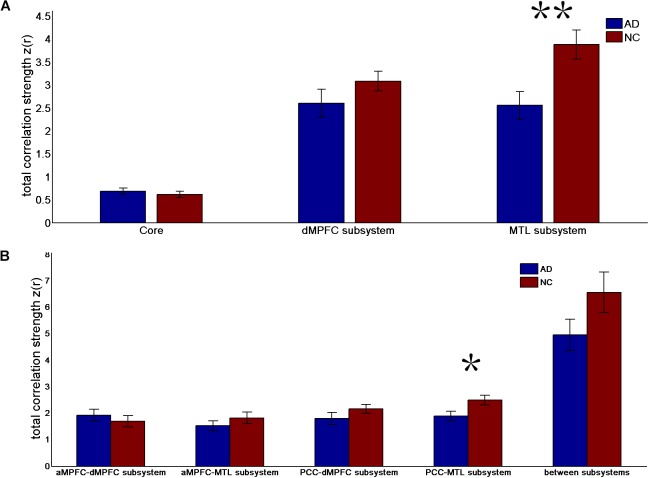
Total correlation strength (positive-only) in the default network subsystems in the control and AD groups. **(A)** Total correlation strength within the core and subsystems of the two groups. **(B)** Total correlation strength between core regions (including the aMPFC and PCC) and subsystems separately and between the two subsystems in the two groups (^∗^*p* < 0.05, ^∗∗^*p* < 0.01). Error bars represent the standard error of the mean.

**FIGURE 2:**
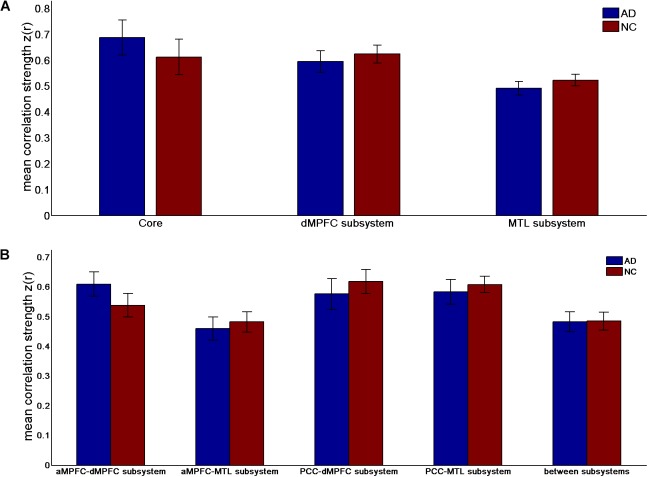
Mean correlation strength (positive-only) in the default network subsystems in the control and AD groups. **(A)** Mean correlation strength within the core and subsystems in the two groups. **(B)** Mean correlation strength between core regions (including the aMPFC and PCC) and subsystems separately and between the two subsystems in the two groups. Error bars represent the standard error of the mean.

**FIGURE 3:**
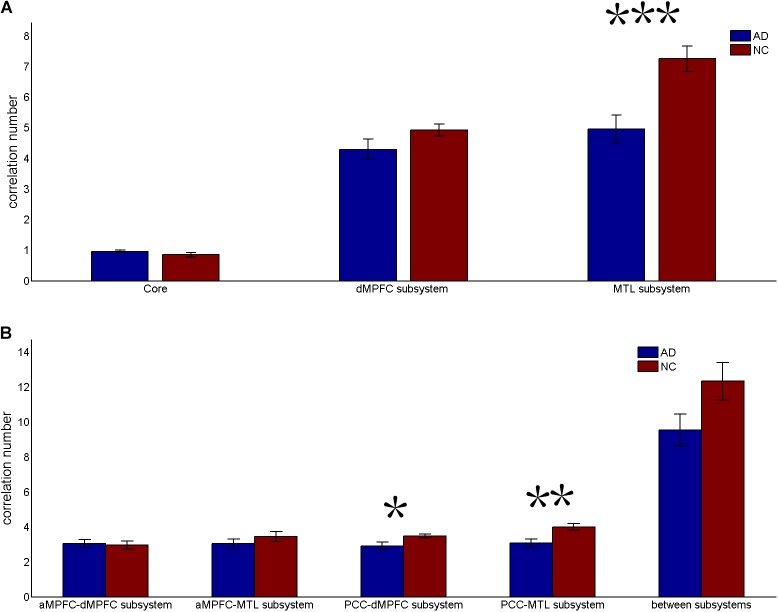
Correlation number (positive-only) in the default network subsystems in the control and AD groups. **(A)** Correlation number within the core and subsystems in the two groups. **(B)** Correlation number between core regions (including the aMPFC and the PCC) and subsystems separately and between the two subsystems in the two groups (^∗^*p* < 0.05, ^∗∗^*p* < 0.01, and ^∗∗∗^*p* < 0.001). Error bars represent the standard error of the mean.

The results of the former method are as follows: Patients with AD showed decreased total FC strength (FCS) in the two subsystems (see Figure 1A) between core regions and subsystems and between subsystems relative to the controls (see Figure 1B). Two-sample *t*-tests were performed to statically analyze the differences between the AD group and the control group. Significant differences between the two groups were found in the MTL subsystem (*t*(49) = −3.00, *p* = 0.004) but not in the core (*t*(49) = 0.77, *p* = 0.45) or the DMPFC subsystem (*t*(49) = −1.31, *p* = 0.20). Significantly decreased total FCS was also found between the PCC and the MTL subsystem (*t*(49) = −2.41, *p* = 0.02) but not found between the subsystems (*t*(49) = −1.64, *p* = 0.11), between the PCC and the dMPFC subsystem (*t*(49) = −1.33, *p* = 0.19), between the amPFC and the dMPFC subsystem (*t*(49) = 0.77, *p* = 0.44), or between the amPFC and the MTL subsystem (*t*(49) = −1.01, *p* = 0.32). For total FCS in the core, stronger correlations were observed in the AD group, although not significant (see Figure 1A). However, there was no significant difference in the mean strength of the FC of the inter- and intra-subsystems (see Figures 2A,B) between the two groups. For the FC matrix of each subject, we also calculated the total number of intra- and inter-subsystem FCS (see Figure 3). The statistical analysis (two-sample *t*-tests) revealed that the AD group showed a significantly smaller total number of FCS in the MTL subsystem (*t*(49) = 3.74, *p* = 0.0005) (see Figure 3A). Moreover, a significantly reduced number of FCS was also found between the PCC and the MTL subsystem (*t*(49) = 3.12, *p* = 0.003) and between the PCC and the DMPFC subsystem (*t*(49) = 2.47, *p* = 0.02) (see Figure 3B) in AD patients relative to controls.

**FIGURE 4:**
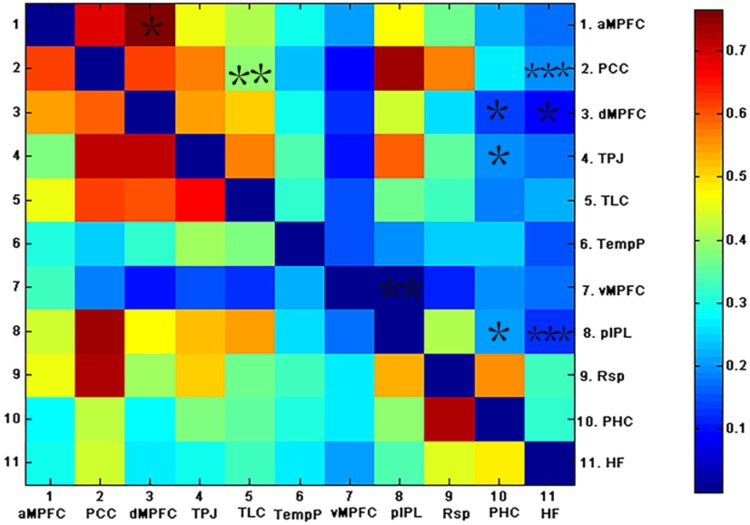
The above matrices show the correlation coefficients between the ROIs in the DMN, as well as the group differences (^∗^*p* < 0.05, ^∗∗^*p* < 0.01, and ^∗∗∗^*p* < 0.001). The bottom and top triangles represent the group average correlation matrix of the control and AD groups, respectively. The color bar indicates the correlation coefficients between regions.

### Group Differences in the Inter-Regional FCS of the DMN

To examine which pairs of brain regional correlation coefficients contributed to the alteration in the subsystems of the DMN, correlation coefficients between pairs of ROIs in the DMN for each subject were compared across groups (see Figure 4). As shown in Figure 4, weaker correlations between ROIs in the DMN were observed in the AD group relative to the control group. Fewer inter-regional FCS were found within the MTL subsystem of AD patients than within that of controls: pIPL showed low FC with the hippocampus, the PHC and the vMPFC. In addition, fewer pairs of brain regional FCS of the inter-subsystems were found in AD patients. The hippocampus and the PHC showed decreased FC with the TPJ and the dMPFC, both of which were located in the DMPFC subsystem and the PCC. The PCC also showed decreased FC with the TLC, which exists in the dMPFC subsystem in AD. The functional connection between the hippocampus and the PCC is the only connection that was retained after FDR correction.

**FIGURE 5:**
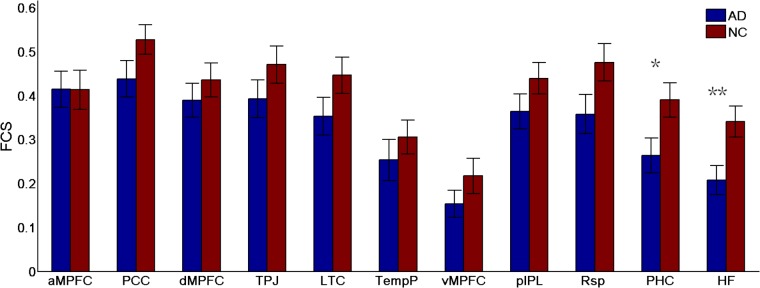
Regional functional connectivity strength (FCS) in the control and AD groups (^∗^*p* < 0.05, ^∗∗^*p* < 0.01). Error bars represent the standard error of the mean.

### The Most Significantly Disrupted Regions Measured by FCS

To identify which regions made the strongest contributions to the changes in subsystems of the DMN, the differences in the regional FCS between the two groups were measured (see Figure 5). Different analysis strategies consistently produced the same results. Here, we present the results of the positive-only strategy. Between-group comparison analysis (two-sample *t*-tests) revealed that regions showing the most significant group differences in FCS were the HF (*t*(49) = 2.76, *p* = 0.008) and the PHC (*t*(49) = 2.28, *p* = 0.03).

## Discussion

Our results suggest that the memory-related MTL subsystem is predominantly disrupted in AD. First, we explored the changes in FC of the intra- and inter-subsystems in patients with AD. The strength and number of FCS were significantly decreased, specifically in the MTL subsystems but not in the core or the DMPFC subsystem. Moreover, the strength and number of FCS were significantly decreased between the MTL subsystems and the PCC. We also examined which pairs of brain regional functional connections contributed to the changes in the subsystems and found that the HF and the PHC showed extensively decreased FCS with other regions in the DMN. Furthermore, we identified the HF and the PHC as making the strongest contributions to the alterations in the subsystems. Additionally, our data show that in addition to the changes in the MTL subsystem, the core and DMPFC subsystems in the default network also have some degree of disruption. A significantly decreased number of FCS between the core and the DMPFC subsystem was found in AD patients. Inter-regional FC analysis revealed that there are a few pairs of decreased brain regional FCS between the subsystems and between the core and the dMPFC in patients with AD.

### Predominantly Disrupted Memory-Related Subsystems in Patients With AD

As consistently reported in the literature, the MTL subsystem is reliably activated when engaged in autobiographical memory and episodic future thought (Andrews-Hanna et al., 2014). These spontaneous thoughts may allow individuals to construct and simulate scenarios, mentally organize their plans, and prepare for what may lie ahead (Immordino-Yang et al., 2012). Additionally, spontaneous thoughts may facilitate consolidation of the most self-relevant information into long-term memory. In the present study, we found that the MTL subsystem is predominantly disrupted. Within the MTL subsystem, the PIPL showed decreased FC with the HF, the PHC and the vMPFC. This finding was supported by the research of Wang L. et al. (2006) and Wang Z. et al. (2013). The HF and the PHC located in the MTL subsystem were mostly disrupted in AD patients. Consistent with our findings, previous researchers have proposed that pathological amyloid depositions and atrophy in AD patients are located in cortical regions such as the HF and the PHC (Buckner et al., 2005, 2009; Stricker et al., 2012). fMRI studies have revealed that AD patients have reduced activity in regions of the DMN. Greicius et al. (2004) found decreased resting-state activity in the hippocampus of AD patients. The PHC and the HF possibly play significant roles in the MTL subsystem because the cognitive processes in which the MTL subsystem is involved might be supported by the MTL (including the PHC and the HF), which dynamically interacts with cortical regions within and outside the default network. Recent studies have supported the possibility of resting MTL activity or MTL-cortical FC related to individual differences in spontaneous episodic thoughts (Andrews-Hanna et al., 2010a), memory ability (Wig et al., 2008), and the consolidation of recent experiences (Tambini et al., 2010). Thus, the changes in the FC of the HF and the PHC in the DMN probably reflect cognitive impairment in recent episodic memory. Our findings support the hypothesis that the memory-related MTL subsystem of the DMN is primarily disrupted in AD.

In addition to the changes within the MTL subsystem itself, in the present study, we found that the MTL subsystem also showed decreased number and strength of FCS with the PCC in patients with AD. A number of studies have also suggested that the PCC plays an essential role in spatial orientation, self-appraisal and internal monitoring as well as memory processing (Gusnard and Raichle, 2001; Greicius et al., 2003; Whishaw and Wallace, 2003; Ries et al., 2006). As the core of the DMN, the PCC, which exhibits strong functional coherence with both subsystems (Andrews-Hanna et al., 2014) in coordination with the MTL subsystems, may facilitate memory processing. As consistently reported in previous literature, in the early stage of AD, FC between the PCC and the hippocampus, the anterior cingulate cortex, the MPFC and the precuneus are disrupted (Greicius et al., 2004; Wang L. et al., 2006; Sorg et al., 2007). It is possible that impairment between the PCC and the MTL subsystem may damage memory processing. These findings indicate that AD predominantly disrupts the memory-related MTL subsystem.

### More Extensive Disruption of Subsystems in Patients With AD

In addition to the predominantly decreased FC associated with the memory-related MTL subsystem, we also found more extensively reduced FC in the inter-subsystems. Compared with the control group, the number of FCS between the PCC and the DMPFC subsystem was significantly reduced in AD, and inter-regional FC revealed that a few pairs of brain regional FCS between the subsystems and between the core region and the dMPFC subsystem exhibited significant decreases in the AD group. In line with our findings, previous studies have found that dissociated FC occurs between the PCC and the inferior temporal cortex in AD patients (Zhang et al., 2010). AD patients also have decreased hippocampus connectivity with the TPJ (Greicius et al., 2004) and the MPFC (Grady, 2001). These results provide powerful evidence for the possibility of the deterioration of all subsystems of the DMN as AD progresses, and previous studies support this possibility. A resting-state FC study suggested that early in the disease, regions of the posterior DMN, including the PCC/Rsp, the IPL, and the LTC (Buckner et al., 2005), begin to disengage their connectivity; however, as the disease progresses, connectivity within all systems eventually deteriorates (Damoiseaux et al., 2012). Zhang et al. (2010) suggested that impairment in the PCC FC changes with AD progression. In contrast to the constructive functions of the MTL subsystem, the DMPFC subsystem may play an important role in examining the mental states of social agents (Andrews-Hanna, 2012), including the ability to reflect on, evaluate, or appraise social information. The PCC shares functional properties with both subsystems, possibly interacting with the DMPFC to facilitate examination of the mental state. The DMN subsystems supporting component processes of self-generated thought work together but independently to complete internal information processing and ultimately guide and motivate behavior. For example, the DMPFC subsystem may allow individuals to reflect on the mental states elicited by the stimulus, and the MTL subsystem may allow individuals to integrate this introspective information into a goal-directed plan (Andrews-Hanna, 2012). Therefore, more extensive disruption of the subsystems of the DMN in patients with AD in the DMN may account for additional cognitive impairments other than memory deficits to some degree. Our findings support previous studies indicating that the DMN is an important target network to be impaired (Buckner et al., 2009) and specify that different subsystems of the default network are not equivalently damaged in AD but that memory-related MTL subsystems are selectively damaged. As the disease progresses, other subsystems of the DMN may also have a certain degree of damage.

### Limitations

Several limitations in the present work should be considered. First, our study had a small sample size. Second, our research focused only on subjects with mild to moderate AD, which did not allow us to determine the specific changes in the DMN subsystems during different stages of the disease. Third, we did not perform related behavioral tests. Specific self-referential cognitive ability changes in AD patients may be related to the corresponding FC in the DMN subsystems. Four, our processing of the functional matrix of each subject may remove some of the weaker connections that are actually present. Considering these limitations, future work should involve a longitudinal study (i.e., from mild cognitive impairment (MCI) to mild, moderate, and serious AD) and include more participants in each group to enhance stability of the results. In addition, we should explore the relationship between the changes in the DMN subsystems in the functional and structural network and specific self-referential cognitive ability. Furthermore, we will explore more reasonable ways to implement our research.

## Conclusion

In summary, this study shows that the memory-related MTL subsystem of the DMN is predominantly disrupted in patients with AD and further that the core and DMPFC subsystems in the DMN are also disrupted to some degree. These findings indicate the different levels of disruption of distinct subsystems of the DMN, possibly providing novel insight into the potential pathogenesis of AD and a possible imaging biomarker for the early diagnosis of AD.

## Ethics Statement

This study was carried out in accordance with the recommendations of “GCP and SFDA, Ethics Review Committee of Huashan Hospital affiliated to Fudan University” with written informed consent from all subjects. All subjects gave written informed consent in accordance with the Declaration of Helsinki. The protocol was approved by the “Ethics Review Committee of Huashan Hospital affiliated to Fudan University.”

## Author Contributions

HQ and XZ designed the study. HQ analyzed and interpreted the data and drafted the manuscript. HQ and HL revised the manuscript and interpreted the data. XZ, HMH, and HJH acquired the data. All the authors read and approved the final manuscript.

## Conflict of Interest Statement

The authors declare that the research was conducted in the absence of any commercial or financial relationships that could be construed as a potential conflict of interest.

## Footnotes

1 http://www.fil.ion.ucl.ac.uk/spm

2 http://www.restfmri.net
